# Validation of Promoters and Codon Optimization on CRISPR/Cas9-Engineered Jurkat Cells Stably Expressing αRep4E3 for Interfering with HIV-1 Replication

**DOI:** 10.3390/ijms232315049

**Published:** 2022-11-30

**Authors:** Koollawat Chupradit, Kanokporn Sornsuwan, Kritayaporn Saiprayong, Methichit Wattanapanitch, Chatchai Tayapiwatana

**Affiliations:** 1 Siriraj Center for Regenerative Medicine, Research Department, Faculty of Medicine Siriraj Hospital, Mahidol University, Bangkok 10700, Thailand; 2 Center of Biomolecular Therapy and Diagnostic, Faculty of Associated Medical Sciences, Chiang Mai University, Chiang Mai 50200, Thailand; 3 Division of Clinical Immunology, Department of Medical Technology, Faculty of Associated Medical Sciences, Chiang Mai University, Chiang Mai 50200, Thailand; 4 Center of Innovative Immunodiagnostic Development, Faculty of Associated Medical Sciences, Chiang Mai University, Chiang Mai 50200, Thailand

**Keywords:** alpha repeat protein, CRISPR/Cas9, promoter, codon optimization, HIV-1

## Abstract

Persistent and efficient therapeutic protein expression in the specific target cell is a significant concern in gene therapy. The controllable integration site, suitable promoter, and proper codon usage influence the effectiveness of the therapeutic outcome. Previously, we developed a non-immunoglobulin scaffold, alpha repeat protein (αRep4E3), as an HIV-1 RNA packaging interference system in SupT1 cells using the lentiviral gene transfer. Although the success of anti-HIV-1 activity was evidenced, the integration site is uncontrollable and may not be practical for clinical translation. In this study, we use the CRISPR/Cas9 gene editing technology to precisely knock-in αRep4E3 genes into the adeno-associated virus integration site 1 (AAVS1) safe harbor locus of the target cells. We compare the αRep4E3 expression under the regulation of three different promoters, including cytomegalovirus (CMV), human elongation factor-1 alpha (EF1α), and ubiquitin C (UbC) promoters with and without codon optimization in HEK293T cells. The results demonstrated that the EF1α promoter with codon-optimized αRep4E3mCherry showed higher protein expression than other promoters with non-optimized codons. We then performed a proof-of-concept study by knocking in the αRep4E3mCherry gene at the AAVS1 locus of the Jurkat cells. The results showed that the αRep4E3mCherry-expressing Jurkat cells exhibited anti-HIV-1 activities against HIV-1NL4-3 strain as evidenced by decreased capsid (p24) protein levels and viral genome copies as compared to the untransfected Jurkat control cells. Altogether, our study demonstrates that the αRep4E3 could interfere with the viral RNA packaging and suggests that the αRep4E3 scaffold protein could be a promising anti-viral molecule that offers a functional cure for people living with HIV-1.

## 1. Introduction

Gene therapy involves correcting defective genes, replacing malfunctioning genes, and adding therapeutic genes. Several factors affect the expression levels of transgenes, including promoters, enhancers, polyadenylation signals, and other expression elements. The promoter is one of the critical factors influencing the expression level of the protein of interest. It is known that each gene has its specific promoter. Some promoters can function in specific cell types called tissue-specific promoters. However, several non-specific promoters, such as human cytomegalovirus (CMV) and human elongation factor-1 alpha (EF1α), are commonly used in gene therapy applications [1,2]. Even though the CMV promoter is a strong promoter, certain studies demonstrated that gene silencing is associated with DNA methylation leading to the downregulation of gene expression [3,4]. The EF1α promoter has also shown high protein expression levels in primary cells and various cell lines [5,6]. Even though the ubiquitin C (UbC) promoter promotes stable eGFP expression in human embryonic stem cells (hESCs), the level is moderate in comparison to the constitutive CMV enhancer/chicken β-actin promoter (CAG) promoter [7,8]. This indicates that the choice of the promoter is essential for transgene expression in specific cell types. Therefore, selecting appropriate promoters for each cell type is essential for gene therapy applications.

Apart from the promoters, codon usage significantly influences protein expression levels. Even though more than one codon can encode the same amino acid, those codons are not used with the same frequencies and are different in every organism. This phenomenon is called the codon usage bias (CUB) [9,10,11]. CUB plays an essential role in several cellular processes, for example, transcription, mRNA stability, translation, and protein folding [12,13]. The genome-wide expression profile in the previous studies demonstrated positive correlations between codon biases and mRNA levels in prokaryotes and lower eukaryotes. The results revealed that the effects of CUB are mainly involved in the transcription process. [14,15,16,17]. Moreover, suboptimal codon usage affected the translation process and mRNA stability [14]. Interestingly, specific cell types, such as immune cells, have distinct codon usage patterns among other immune cell types [18]. Therefore, in the genetic engineering approach, the codon usage of the target gene is usually optimized to increase the protein expression level.

We aim to compare the effects of promoter and codon usage on protein expression of a scaffold protein, i.e., alpha repeat protein (αRep). Our previous studies demonstrated that the αRep4E3 inhibited the RNA packaging process of HIV-1 in the SupT1 cell line using lentiviral transduction [19]. Since the αRep4E3 is proposed for HIV gene therapy, lentiviral random integration of the transgene into the host chromosome may lead to the risks of insertional mutagenesis and oncogenesis [20]. Recently, the CRISPR/Cas9 system became popular in genetic engineering for precisely editing the chromosome at specific sites [21,22]. Several studies have shown that introducing the transgene into the adeno-associated virus integration site 1 (AAVS1) is safe, and the cells could maintain the transgene expression [23,24,25]. We formerly demonstrated that the mCherry gene could be delivered into the *AAVS1* site of human induced pluripotent stem cells (hiPSCs). The transduced iPSCs expressed high levels of the transgene and exhibited a normal karyotype after the gene editing [26].

In this study, we introduced an anti-HIV gene, αRep4E3mCherry, using the CRISPR/Cas9 technology targeting the *AAVS1* locus. The levels of αRep4E3 expression under the control of three constitutive promoters, including CMV, human EF1α, and UbC promoters, along with different codon optimizations, were first validated in HEK293T cells by plasmid vector transfection. Then, a suitable promoter with modified codons of the αRep4E3mCherry was validated for the effectiveness of αRep4E3 in interfering with HIV-1 propagation in the Jurkat cell line. The coherence of promoter usage and codon optimization shall be considered when sustainable gene therapy in a specific target cell is applied.

## 2. Results

### 2.1. Expression of the αRep4E3mCherry Protein in HEK293T Cells under Various Constitutive Promoters

**Figure 1:**
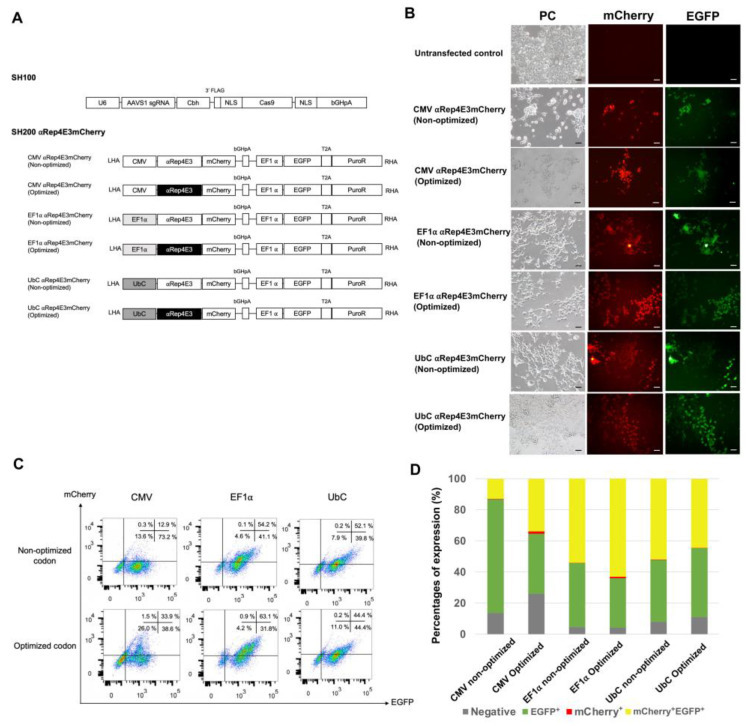
Validation of the αRep4E3mCherry gene constructs for stable gene expression in the HEK293T cells. (**A**) Schematic diagram of CRISPR/Cas9 plasmid vectors, including SH100 encoding the AAVS1 single-guide RNA and Cas9, and SH200 plasmids encoding the αRep4E3mCherry gene with or without codon optimization under the control of CMV, EF1α, and UbC promoters. The downstream of the plasmid vector harbors EGFP and puromycin-resistant genes, LHA = left homology arm, and RHA = right homology arm. (**B**) Representative images of the αRep4E3mCherry expression in the HEK293T cells after puromycin selection for 13 days, PC = phase contrast, scale bar = 200 µm. (**C**) Flow cytometric analysis of mCherry and EGFP expression. The HEK293T cells stably expressing αRep4E3mCherry were harvested on day 19 after puromycin selection. (**D**) The percentages of mCherry and EGFP expression in the HEK293T cells were analyzed by flow cytometry.

We utilized the CRISPR/Cas9 technique to introduce the anti-HIV-1 scaffold gene into HEK293T cells to generate stable cell lines expressing αRep4E3mCherry under various constitutive promoters. Six SH200 plasmid vectors were constructed to use as donor templates for knock-in. The αRep4E3mCherry gene construct was placed under the control of different promoters, including CMV, EF1α, and UbC with or without codon optimization. The mCherry fusion partner was used as a reporter for tracking αRep4E3 gene expression. The downstream genes expressing the green fluorescent protein (EGFP) and puromycin resistance gene were under the control of the EF1α promoter for verifying the transduction efficacy (Figure 1A).

On day 13 after selection, the red and green fluorescent proteins were observed in six versions of the αRep4E3mCherry, as shown in B. Notably, the expression levels determined by the flow cytometer on day 19 demonstrated that the codon-optimized αRep4E3mCherry under the control of CMV and EF1α promoters displayed a significantly higher number of double positive subpopulation compared to the non-optimized codon (Figure 1C, D). Interestingly, the EF1α promoter with codon optimization showed the highest double-positive cells compared to other promoters. These results indicated that the αRep4E3mCherry gene was successfully introduced into the HEK293T cells using the CRISPR/Cas9 technique, and the EF1α promoter with the αRep4E3 optimized codon resulted in the highest expression of αRep4E3mCherry.

### 2.2. CRISPR/Cas9-Mediated αRep4E3mCherry Gene Knock-in in the Jurkat Cell Line

**Figure 2:**
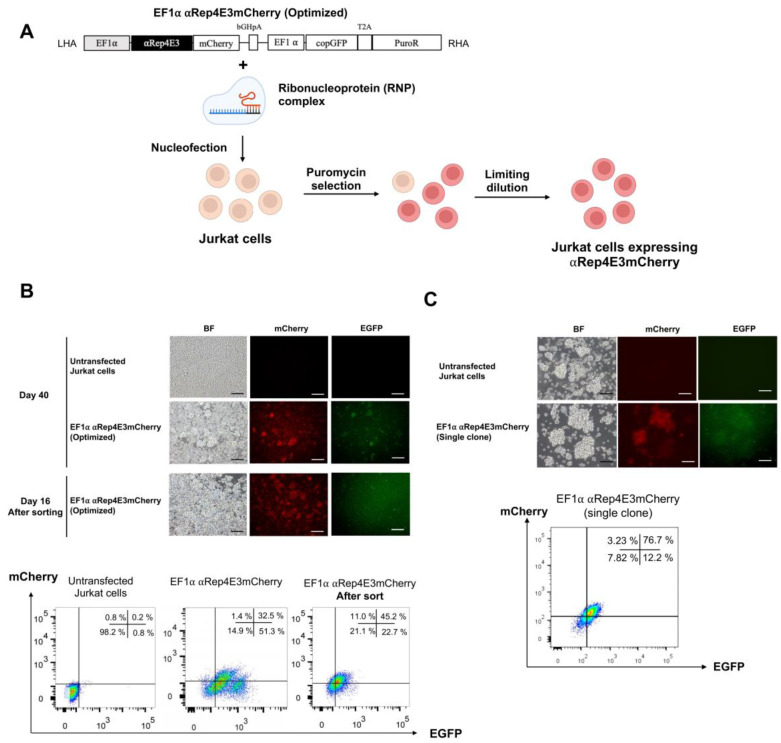
Stable αRep4E3mCherry expression and clonal validation in the Jurkat cells. (**A**) Schematic diagram of the CRISPR/Cas9-based gene delivery targeting the AAVS1 locus using the SH200 donor plasmid and the RNP complex in the Jurkat cells. (**B**) Representative images of the Jurkat cells stably expressing αRep4E3mCherry (optimized codon) under the control of the EF1α promoter after puromycin selection for 40 days and after sorting for 16 days, BF = bright field, scale bar = 200 µm (top panel); flow cytometric analysis of the Jurkat cells expressing αRep4E3mCherry (bottom panel). (**C**) A single clone of the Jurkat cells stably expressing αRep4E3mCherry, scale bar = 100 µm (top panel), and flow cytometric analysis of mCherry and EGFP expression (bottom panel).

We selected the SH200 plasmid DNA containing the EF1α promoter with the αRep4E3 optimized codon for further experiments in the Jurkat cell line due to its highest expression in the HEK293T cells (Figure 2A). On day 40 after puromycin selection, 32.5% of the Jurkat cells were double-positive for mCherry and EGFP, and 51.3% of the Jurkat cells were single positive for EGFP. We then FAC sorted the double-positive cells and cultured them for more than 15 days. The percentage of the double-positive cells increased slightly to 45.2% (Figure 2A,B). We performed limiting dilution to select an mCherry-positive clone. After clonal selection, the percentage of the double-positive population (mCherry^+^ EGFP^+^) increased to 76.7% (Figure 2C).

### 2.3. Analysis of the αRep4E3mcherry Transgene Integration in the Jurkat Clone by Multiplex PCR

**Figure 3:**
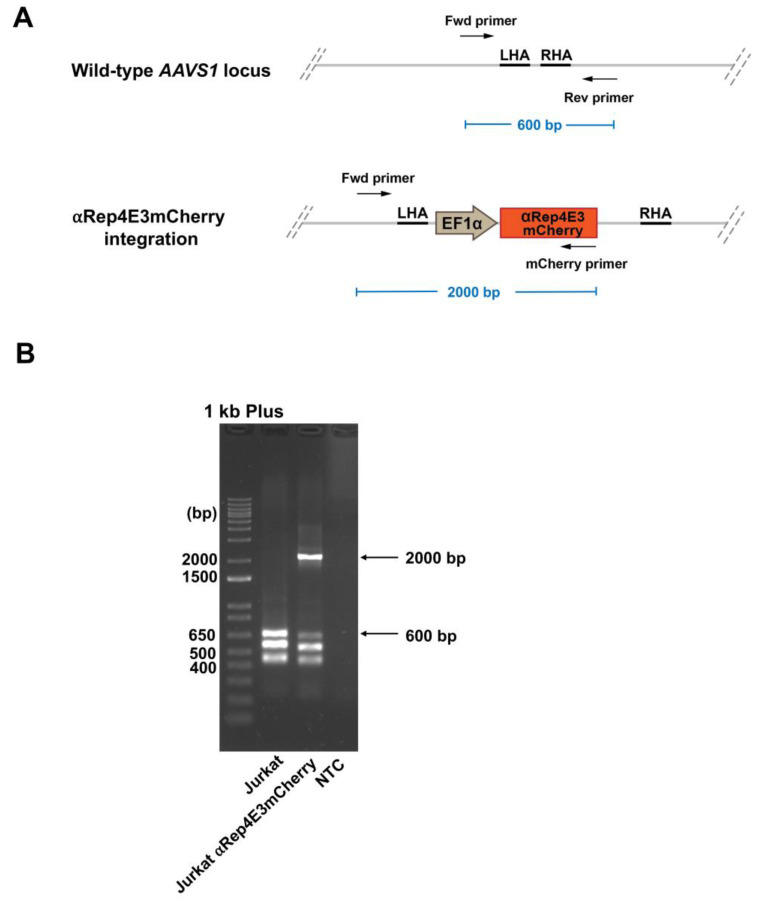
Analysis of αRep4E3mcherry transgene integration in the Jurkat clone by multiplex PCR. (**A**) Schematic diagram of the multiplex PCR amplification. (**B**) The PCR products amplified from the genomic DNA extracted from the untransfected Jurkat cells and the Jurkat cells expressing αRep4E3mCherry, and non-template control (NTC). The 2000-bp band indicates the integrated αRep4E3mCherry gene at the AAVS1 locus, while the 600-bp band represents the wild-type AAVS1 locus.

To confirm the transgene integration after knock-in, we performed a multiplex PCR analysis of the genomic DNA from the Jurkat clone using the primers that amplify the upstream region of the AAVS1 LHA, mCherry reporter protein, and downstream region of the AAVS1 RHA as indicated by the black arrow (Figure 3A). The results demonstrated a successful transgene knock-in at the AAVS1 safe harbor locus on human chromosome 19, as indicated by the 2000-bp PCR product (Figure 3B). However, the lower band (600 bp) was also observed. This result indicated that the αRep4E3mCherry gene was integrated into one allele (monoallelic-edited).

### 2.4. The αRep4E3mCherry Jurkat Clone Conferred Resistance to HIV-1 Infection

**Figure 4:**
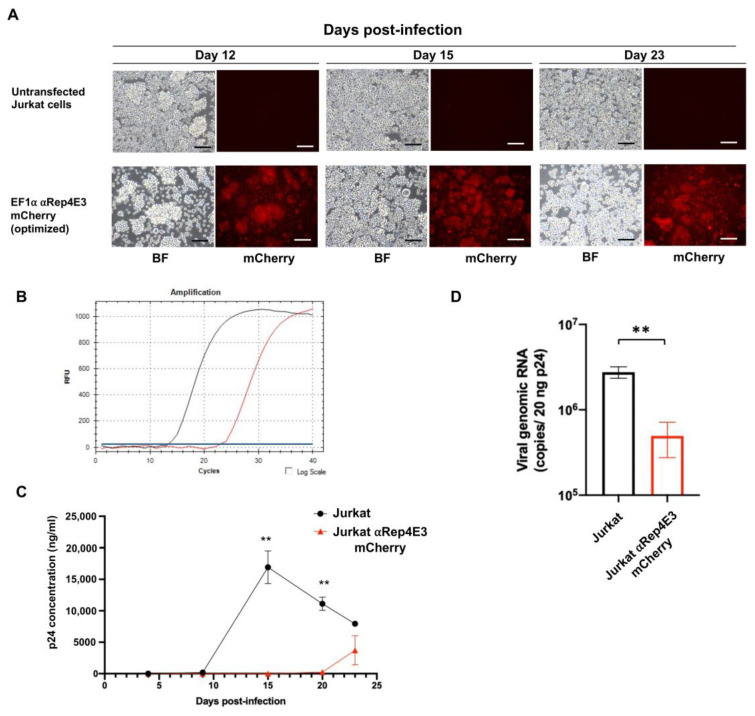
Inhibition of HIV-1 replication in the αRep4E3mCherry-expressing Jurkat cells. (**A**) Morphology and mCherry expression were monitored by light and fluorescence microscopy using an inverted fluorescence microscope, BF = bright field, scale bar = 100 µm. (**B**) The *Alu-gag* quantitative real-time polymerase chain reaction (PCR) (*Alu-gag* qPCR) was performed in the untransfected (black line) and αRep4E3mCherry (red line) expressing Jurkat cells on day 15 pi to determine the HIV-1 integration. (**C**) The culture supernatants were collected at 3-day intervals, from day 5 to day 23 pi. The culture supernatants were assayed for p24 protein. (**D**) HIV RNA copies were determined per 20 ng of p24 protein on day 23 pi using the HIV viral load assay. The bar graph represents the mean ± SD from triplicate wells. ** *p* ≤ 0.01 using unpaired *t*-test.

To evaluate the protection levels of the Jurkat clone stably expressing αRep4E3mCherry against HIV-1, the untransfected and αRep4E3mCherry Jurkat cells were challenged with HIV-1NL4-3 at an MOI of 10. From day 15 post-infection (pi) onwards, the untransfected Jurkat cells displayed multinucleated giant cells, a sign of viral infection, slightly more than the αRep4E3mcherry Jurkat cells. However, the αRep4E3mCherry Jurkat cells still expressed mCherry reporter protein until day 23 pi (Figure 4A). The Alu-gag PCR on day 15 pi showed a delayed Cq of 22.95 in the αRep4E3mcherry Jurkat cells compared to 13.36 in the control Jurkat cells, indicating that the Jurkat cells stably expressing αRep4E3mCherry exhibited lower HIV-1 integration compared to the control Jurkat cells (Figure 4B). The levels of anti-viral protection were evaluated in the culture supernatants collected on days 5, 9, 15, 20, and 23 pi using an ELISA. Following HIV-1 infection, high levels of p24 protein were observed in the culture supernatant from the control Jurkat cells on day 15 pi, while the levels of p24 protein released from the αRep4E3mcherry Jurkat cells were suppressed until day 20 pi (Figure 4C). The result from the Alu-gag PCR was concordant with the ELISA results, indicating that the αRep4E3 protein initially delayed HIV-1 infection in the Jurkat cells. We further investigated the role of the αRep4E3 protein in RNA packaging interference following our published protocol [19]. The incorporated viral RNA was quantified using the viral load assay. Our results revealed that at the equal concentration of 20 ng/mL p24 protein on day 23 pi, the viral genomic RNA in the culture supernatant harvested from the αRep4E3mCherry Jurkat cells was significantly lower than those of the control Jurkat cells (Figure 4D). This finding reflects an anti-HIV-1 activity of the αRep4E3 protein at the RNA packaging process, although a slight increase in the level of p24 protein was observed on day 23 pi.

## 3. Discussion

Gene therapy is an approach to treat patients with both inherited and acquired diseases by genetically modifying patients’ cells. Viral vectors are widely used since they rapidly invade target cells using their natural ability. However, random integration can lead to the risk of genotoxicity [27,28]. Previously, we delivered the gene encoding αRep4E3 controlled by the MND promoter into the SupT1 cells using lentiviral transduction. Even though the results showed stable protein expression and anti-HIV-1 activity, the transgene copy number and integration site were not verified [19]. These factors could probably bias the data interpretation. CRISPR/Cas9 has become a versatile tool for gene editing due to low off-target activity, ease of use, and the ability to perform multiplex gene editing [29]. Furthermore, the advantages of CRISPR/Cas9 are high controllability and the ability to identify the gene editing sites correctly. The previous study showed that CRISPR/Cas9 targeting CXCR4 or CCR5 chemokine receptors, the co-receptors for HIV-1, protected the primary CD4^+^ T cells from viral infection [30]. Moreover, using a single-guide RNA (sgRNA) targeting the long terminal repeat (LTR) sequence or other essential genes for viral replication could inactivate the HIV-1 provirus by introducing a mutation into the target region [31]. However, the leakage of the virus from using one sgRNA still occurred. Thus, molecules that bind to the conserved regions could overcome this limitation.

Several attempts have been made to inhibit HIV-1 infection. Our previous studies demonstrated the use of scaffold proteins, including ankyrin repeat protein, zinc finger protein, and αRep4E3 protein, as anti-HIV-1 molecules. The results showed that these designed scaffold proteins could inhibit HIV-1 infection at various steps of the viral life cycle [19,32,33]. The present study used the αRep4E3 targeting the downstream conserved region of the zinc finger domain in the nucleocapsid (NC). The binding of the αRep4E3 to the NC results in the inhibition of the HIV-1 RNA packaging process, in which the viral RNA molecules are recognized by the nucleocapsid (NC) domain during the viral assembly [19]. Previous studies showed that the CHCC residues of the NC zinc fingers are highly conserved, and the mutation of this region resulted in the production of non-infectious viruses [34,35,36,37]. Hence, using the αRep4E3 protein for HIV-1 treatment may alleviate the probability of viral escape.

Since the promoter choice affects the protein expression levels, we first screened different constitutive promoters, including CMV, EF1α, and UbC, with and without codon optimization in the HEK293T cells. All the transfected HEK293T cells expressed mCherry protein after antibiotic selection. Interestingly, the EF1α promoter resulted in the highest percentages of double-positive cells, followed by the UbC and CMV promoters, indicating that the transgene promoter significantly influences the gene expression. Our result contradicts the previous study that HEK293T cells expressed the highest level of EGFP integrated at the *AAVS1* locus under the CMV promoter, followed by EF1α, SV40, and TK promoters [38]. Apart from the promoter, the secondary structure of the transcribed RNA influences individual protein expression levels [39]. Fine-tuning the coding sequence to optimize mRNA structure should be further considered [40]. 

In addition to the promoter choice, a significant difference in protein expression was observed after codon optimization in the HEK293T cells. This result implied that CUB also affects protein expression. Several studies showed that codon changes could result in changes in the conformation and stability of the protein [41,42]. Codon optimization is an algorithm designed to increase the expression of the protein of interest by changing the base for synonymous codons. Some studies demonstrated that optimizing codon usage bias could enhance the recombinant protein expression in different host organisms [43,44,45]. The crucial codon bias is evident in specific cells, such as monocytes, B and T lymphocytes. Previously the mutational bias influenced the codon bias in monocytes, while the translational selection could affect codon usage in B and T cells [18]. Thus, optimization for T cell codon usage should benefit the expression of the Rep4E3mCherry in Jurkat cells.

To determine whether codon usage affects protein expression, we compared the non-codon and codon-optimized αRep4E3 protein expression. The results showed that the codon-optimized αRep4E3mCherry HEK293T cells had higher mCherry^+^EGFP^+^ expression than the non-codon optimization in the CMV and EF1α promoters. However, there was no difference in the double-positive cells in the non-codon and codon-optimized αRep4E3 under the UbC promoter. Hence, the EF1α promoter with the codon-optimized αRep4E3mCherry was chosen for further experiments in the Jurkat cells. The Jurkat cells expressing αRep4E3mCherry showed anti-HIV activity when infected with HIV-1_NL4-3_. The increase in the p24 level in the αRep4E3mCherry Jurkat cells was observed on day 23 pi, indicating that the virus could assimilate viral particles in the engineered cells after a certain period. However, the infectivity of these particles is lower since most of them are HIV genome free suggesting that the αRep4E3 functions in interfering with the RNA packaging process. This result is consistent with our previous study, in which lentiviral transduction was applied to generate the SupT1 cells expressing the αRep4E3 protein. In addition, monoallelic expression of the αRep4E3mCherry gene was adequate to interfere with HIV-1 replication. After day 20, the p24 level was saturated and beyond the interference efficacy of the αRep4E3mCherry protein. The load of HIV-1 in the culture supernatant is much higher than the median viral loads in HIV-1-infected individuals [46]. Thus, the HIV-1 protection by αRep4E3mCherry should be further validated to ensure its clinical significance.

In conclusion, we successfully delivered the αRep4E3mCherry gene into the *AAVS1* locus and explored the effects of the promoter and codon usage of αRep4E3mCherry gene in HEK293T cells. The results demonstrated that the EF1α promoter with the codon-optimized αRep4E3 showed the highest protein expression among other promoters. In addition, the Jurkat stably expressing the αRep4E3mCherry protein exhibited anti-HIV-1 activity by inhibiting the viral RNA packaging process resulting in the reduction of viral genome copies. These findings will be a foundation for establishing engineered stem cells for HIV gene therapy.

## 4. Materials and Methods

### 4.1. Cells

Human embryonic kidney (HEK293T) cells and Jurkat cells, Clone E6-1 (a human T lymphoblast cell line susceptible to HIV-1 infection) were obtained from the ATCC (Manassas, VA, USA). HEK293T cells were cultured in Dulbecco’s modified Eagle’s medium (DMEM) (Gibco, Invitrogen, Grand Island, NY, USA), and Jurkat cells were maintained in RPMI-1640 medium, both supplemented with 10% fetal bovine serum (FBS) (Gibco, Invitrogen), 2 mM GlutaMax (Gibco, Invitrogen), and 100 units/mL of penicillin-streptomycin (Gibco, Invitrogen). All cells were maintained in a 37 °C humidified incubator containing 5% CO_2_. The cells were passaged every 2–3 days at a subcultivation ratio of 1:5–1:6.

### 4.2. Optimization of Codon Usages

We optimized the codon of the αRep4E3 gene using GenSmart Codon Optimization (GenScript) by inserting the αRep4E3 gene into the website and selecting a human-T cell as a host organism. The optimized αRep4E3 DNA sequences were synthesized by Integrated DNA Technologies. Both the non-optimized and optimized codons were used for the SH200 donor plasmid (GeneCopoeia, Rockville, MD, USA) construction.

### 4.3. Construction of the Donor Plasmid Vector Harboring αRep4E3mCherry Gene

The CRISPR/Cas9 system used in this study is a plasmid-based method consisting of the SH100 plasmid (GeneCopoeia) carrying sgRNA targeting the *AAVS1* locus and Cas9 gene, and the SH200 plasmid carrying *AAVS1* left homology arm (LHA), different promoters including CMV, UbC or EF1α with either codon-optimized or non-codon-optimized aRep4E3 sequence, mCherry, EGFP/puro, and *AAVS1* right homology arm (RHA) (Figure 1A). The non-codon-optimized aRep4E3 sequence was obtained from our previous work [19]. The optimized αRep4E3 DNA sequences were synthesized by Integrated DNA Technologies. The αRep4E3mCherry gene was then amplified and ligated into the SH200 plasmid vector using *Xba*I and *BstB*I restriction enzyme digestions to obtain the SH200 αRep4E3mCherry donor plasmid vector. The DNA sequences of promoter and αRep4E3mCherry genes were confirmed by DNA sequencing.

### 4.4. CRISPR/Cas9 αRep4E3mCherry Gene Delivery and Expression in Cell Lines

We first compare the expression of the αRep4E3mCherry gene in HEK293T cells using a donor plasmid with different types of promoters and codon usage. The HEK293T cells were transfected with a 1:1 ratio of the SH100 plasmid (GeneCopoeia) encoding the AAVS1 sgRNA and Cas9 protein, and the SH200 plasmid DNA harboring αRep4E3mCherry gene. Briefly, 1 µg of SH100 and 1 µg of SH200 plasmid DNA were co-transfected into 2 × 10^5^ HEK293T cells using Lipofectamine 3000 Reagent (Thermo Fisher, Waltham, MA, USA). The transfected cells were cultured at 37 °C, 5% CO_2._ Two days post-transfection, the transfected HEK293T cells were treated with 200 ng/mL of puromycin. The puromycin concentration was gradually increased to 500 ng/mL for enrichment after 4 days. The puromycin selection was carried out until all the untransfected control cells died.

The SH200 plasmid DNA that gave the highest αRep4E3mCherry expression level was selected for further experimentation in the Jurkat cells. Gene knock-in was carried out using the ribonucleoprotein (RNP) complex. Briefly, 150 µg/mL of Alt-R^®^ S.p. Cas9 Nuclease V3 (Integrated DNA Technologies, Coralville, IA, USA) was mixed with 90 µg/mL of sgRNA targeting the *AAVS1* site generated by GeneArt™ Precision gRNA Synthesis Kit (Invitrogen), and incubated at room temperature for 10 min. 

The Jurkat cells were transfected with the RNP complex using Lonza P3 Primary Cell 4D-Nucleofector X Kit S (Lonza, Basel, Switzerland). Briefly, 5 × 10^5^ Jurkat cells were resuspended with 20 µL of P3 4D-Nucleofector solution containing preincubated RNP and 3.6 µg of SH200 plasmid DNA using CL120 protocol by a 4D-Nucleofector X unit. The transfected cells were incubated in a complete RPMI-1640 medium at 37 °C, 5% CO_2_ for 16–18 h. After incubation, 100 ng/mL of puromycin was added to the culture medium to select the transfected cells.

### 4.5. Fluorescence-Activated Cell Sorting (FACS)

After puromycin selection, the Jurkat cells stably expressing αRep4E3mCherry were expanded for cell sorting—1 × 10^7^ cells were resuspended in 2%FBS/PBS. The cell sorting was performed by a flow cytometer (FACSAria III, BD Biosciences, Franklin Lakes, NJ, USA). The sorted pool was then cultured in a complete RPMI-1640 medium supplemented with 200 units/mL of penicillin-streptomycin. 

### 4.6. Clonal Isolation by Limiting Dilution

The αRep4E3mCherry-sorted pool was resuspended in a complete RPMI-1640 medium to obtain a seeding density of 4 cells/100 µL/well. Then 100 µL of the cell suspension was transferred to each well of the 96-well flat-bottom plate and incubated at 37 °C, 5% CO_2_. A single colony was selected and expanded for further experiments.

### 4.7. Determination of the αRep4E3mCherry Integration Site in the Knock-in Cell Lines

The genomic DNA of the untransfected Jurkat cells and the Jurkat cells stably expressing αRep4E3mCherry was extracted using the High Pure PCR Template Kit (Roche, Basel, Switzerland). Twenty-five nanograms of genomic DNA was used as a template for PCR using primers specific to the left homology arm of the *AAVS1* locus and *mCherry* gene. The primer sequences were: Fwd AAVS1 primer, 5′-TCTTGTAGGCCTGCATCATCACC-3′, Rev AAVS1 primer, 5′-AGGATCCTCTCTGGCTCCAT-3′, and mCherry primer, 5′-TCGTAGGGGCGGCCCTCGC-3′. The reactions were performed in the final volume of 25 µL using DreamTaq Green PCR Master Mix (2X) (Thermo Fisher) using the following program: 20 s at 95 °C, followed by 35 cycles of denaturation at 95 °C for 3 s and annealing at 70 °C for 30 s and extension at 72 °C for 2 min and 10 s.

### 4.8. Viral Stocks

Replication-competent HIV-1_NL4-3_ virus (X4-tropic) was produced by transfecting the pNL4-3 plasmid into HEK293T cells. Briefly, 3.5 × 10^6^ HEK293T cells were seeded onto 10-cm dishes and transfected with 5 µg of the pNL4-3 plasmid, using Lipofectamine 3000 reagent (Thermo Fisher), as previously described [33]. After incubation for 5 h, the transfection mixture was removed, replaced with 10 mL of complete medium, and incubated at 37 °C, 5% CO_2_. The HIV-1 virus was harvested from the culture supernatants by filtration through sterile syringe filters with a Millex-HA syringe filter unit, 0.45-µm pore size (Merck, Rahway, NJ, USA). HIV-1 samples were aliquoted and kept frozen at −80 °C. The culture supernatants were subjected to HIV viral load assay. The level of HIV virion was evaluated using reverse transcription quantitative polymerase chain reaction (RT-qPCR) by COBAS Ampliprep/COBAS Taqman HIV-1 test (Roche).

### 4.9. HIV-1 Infection

To determine the anti-viral protection of αRep4E3mCherry, the Jurkat cells expressing αRep4E3mCherry were challenged with the X4-tropic HIV-1NL4-3. The untransfected Jurkat cells and the Jurkat cells expressing αRep4E3mCherry were maintained in a growth medium for at least 4 weeks prior to the HIV-1 challenge. The cells were incubated with HIV-1 at a multiplicity of infection (MOI) of 10 for 16 h. The cells were then washed three times with a serum-free medium and resuspended in a fresh growth medium. They were split (1:2) at 3-day intervals to maintain a cell density of approximately 10^6^ cells/mL. HIV-1 replication was monitored in the culture supernatant by enzyme-linked immunosorbent assay (ELISA) using Genscreen ULTRA HIV Ag-Ab (Biorad, Hercules, CA, USA) by capsid (p24) protein detection and viral load assay described above. The cell pellets were kept for determining the HIV-1 integration using the *Alu-gag* PCR. To further determine the HIV-1 viral RNA, the culture supernatants on day 23 post-infection (pi) were subjected to HIV viral load assay. The levels of HIV virion in the culture supernatant were normalized to 20 ng/mL of p24 protein. The viral load was determined by RT-qPCR by COBAS Ampliprep/COBAS Taqman HIV-1 test (Roche).

### 4.10. HIV-1 Integration Assay

The HIV-1 integration was determined by a conventional *Alu-gag* qPCR assay [47,48]. Briefly, the genomic DNA of the HIV-1 infected cells was extracted using the High Pure PCR Template Kit. The first round of PCR was performed using a pair of specific primers against *Alu* (human) and *gag* (HIV-1) sequences. The primer sequences were: *Alu* forward, 5′-GCC TCC CAA AGT GCT GGG ATT ACA G-3′, and HIV-1 *gag* reverse, 5′-GTT CCT GCT ATG TCA CTT CC-3′. The reactions were performed in the final volume of 25 µL using DreamTaq Green PCR Master Mix (2X) (Thermo Fisher). The second round of RU5 kinetic PCR was performed using 10 µL of undiluted first-round PCR product. The primer sequences were U3_FWD_SS_RIHES01, 5′- AGT GGA GGT TTG ACA GCC GCC TA -3′ and deltaU3_REV_SS_RIHES, 5′-CCA GTA CAG GCA AAA AGC AGC TGC-3′. The deltaU3 molecular probe, labeling at the 5′ terminus with the 6-carboxyfluorescein (FAM) reporter and at the 3′ terminus with the Black Hole Quencher (BHQ), contains the following sequence: 5′-6FAM- CCT CCC TGG AAA GTC CCC AGC GGA AAG TCC-BHQ-3′. The 25-µL reaction volume contained THUNDERBIRD^®^ Probe qPCR Mix (Toyobo, Osaka, Japan), 400 nM of U3_FWD_SS_RIHES01 primer, 400 nM of deltaU3_REV_SS_RIHES primer, and 140 nM deltaU3 molecular beacon probe. The PCR reactions were performed using the MiniOpticon real-time PCR system using the following program: 20 s at 95 °C, followed by 50 cycles of denaturation at 95 °C for 3 s and annealing and extension at 63 °C for 30 s. Glyceraldehyde-3-phosphate dehydrogenase (GAPDH) was used as an internal control for the DNA quantification in each qPCR assay, using the following GAPDH primer sequences: GAPDH_FWD, 5′-GAA GGT GAA GGT CGG AGT C-3′ and GAPDH_REV, 5′-GAA GAT GGT GAT GGG ATT TC-3′. The GAPDHTM molecular beacon probe contained the following sequence: 5′-FAM-CAA GCT TCC CGT TCT CAG CCT-BBQ-3′. The reactions were carried out as previously described.

### 4.11. Statistical Analysis

Statistical analyses were performed using GraphPad Prism software. Statistical significance was determined by the unpaired *t*-test analysis. We indicated the significance levels as follows: *p* < 0.01 **.
